# Dual burden of Zika and COVID-19 in India: challenges, opportunities and recommendations

**DOI:** 10.1186/s41182-021-00378-0

**Published:** 2021-10-18

**Authors:** Mainak Bardhan, Debolina Pramanik, Rizana Riyaz, Mohammad Mehedi Hasan, Mohammad Yasir Essar

**Affiliations:** 1 Division of Bacteriology, ICMR-National Institute of Cholera and Enteric Diseases, Kolkata, India; 2 Medical College Kolkata, Kolkata, West Bengal India; 3 Kasturba Medical College, Mangalore, Manipal Academy of Higher Education, Karnataka, India; 4 Department of Biochemistry and Molecular Biology, Faculty of Life Science, Mawlana Bhashani Science and Technology University, Tangail, Bangladesh; 5 Kabul University of Medical Sciences, 1001 Kabul, Afghanistan

**Keywords:** Zika, COVID-19, India, Public health

## Abstract

The COVID-19 pandemic has wreaked havoc in the world from last year, and any further insults like Zika virus will surely bring the apocalypse unto us. In July 2021, Zika began spreading in India, mainly in the state of Kerala. Zika infection resembles closely COVID-19 and other arboviral infections, which might lead to delayed and misdiagnosis, further leading to underreporting of cases. Some of the feared complications of Zika include Guillain–Barré syndrome and congenital Zika syndrome leading to microcephaly. Thus, Zika virus disease (ZVD) has significant public health and social impacts. Since the trifecta of infectious diseases (host, agent and environment) are all conducive to the spread of Zika in India, there is a huge risk that ZVD might become endemic in India, which is especially dangerous in the backdrop of this pandemic. This has to be stopped at all costs: the main aspects of which are public health measures, vector control and early diagnosis, especially in case of pregnant women. The diversion of healthcare resources for this pandemic has albeit made this difficult, but we must do our bit if we have to overcome this situation.

Dear Editor,

The world today is grappling to control the SARS-CoV-2 pandemic. The situation is even dreadful in India due to the lack of public health awareness among the population combined with an overburdened healthcare system [1]. There have been 33,915,569 confirmed COVID-19 cases, with 450,127 deaths, reported till 8 October, 2021. Overall, 915,465,826 vaccination doses have been given out thus far [2]. In addition to the present COVID-19 burden, the country is dealing with other infectious disease outbreaks, such as fungal infections like mucormycosis [3, 4]. Due of the overwhelming COVID-19 rise, the appearance of fungal illnesses in many parts of India has placed a significant strain to the country's fragile healthcare system. The rise of several fungal illnesses in India, including the black, white, yellow, and green fungus, has brought to light the country's healthcare system's pre-existing inadequacies and vulnerabilities [5, 6]. Any further impacts, such as the recent reports of Zika virus (ZIKV) cases, would drive the health system to the point of collapse.

Since the inception of the COVID-19 pandemic, the index case of the Zika virus disease (ZVD) was reported in Kerala on July 8, 2021, with the tally rising to 65 on August 03, 2021 [7, 8]. ZIKV belongs to the Flaviviridae species that uses arthropod vectors for transmission. The virus spreads via the bite of Aedes spp. (aegypti and albopictus), sexual contact, blood transfusion as well as blood products and transplacental transmission [9]. ZVD had previously been declared a Public Health Emergency of International Concern on the 28th of January 2016 by the World Health Organization (WHO) [10]. In India, ZVD was first reported in 2017, with significant outbreaks in 2018, which included 159 confirmed cases from Rajasthan, which included 63 pregnant women, 130 from Madhya Pradesh, and one from the state of Gujarat [11].

Currently, the actual numbers might be even higher than the official data because of lack of testing facilities, asymptomatic cases and coinfection with diseases with similar presentation as mentioned below. The possible birth of a superimposed epidemic due to ZVD is to be anticipated and this would wreak havoc on the already exhausted health system. The clinical course of Zika in itself is responsible for many public health repercussions [9]. Zika infection is known to be asymptomatic in most cases, with an incubation period ranging from 3 to 12 days [12]. Moreover, the awareness regarding ZVD is extremely low in comparison with other infectious diseases in the Indian population. This will aid in prolonged community transmission of the ZKV, increasing the caseload. The most common symptoms of Zika are fever, joint and muscle pain, rash, headache, conjunctivitis, nausea, vomiting, and general malaise [12]. The infected were mostly seropositive until day 3 and some have been found seropositive up until day 11 [13]. Most of these symptoms overlap with the clinical presentation of not only SARS-CoV-2 infection, but also other arboviral-mediated diseases like dengue and chikungunya and protozoal infections like that of malaria which are endemic to India.

ZIKV disease is in itself a self-resolving infection. Rare complications include Guillain–Barré syndrome, neuropathy and myelitis [9, 14]. Guillain–Barré syndrome spectrum comprises a spectrum of acute, immune‐mediated demyelinating polyneuropathies mostly occurring as a post-infectious event after certain bacterial and viral infections, including COVID-19 [15]. Thus any patient of GBS should be considered a differential diagnosis for both COVID-19 and Zika infection.

Another devastating effect is if a woman is infected during pregnancy, it may result in congenital Zika syndrome, including microcephaly and other congenital malformations; other complications during pregnancy including preterm birth and miscarriage [14]. This in turn, results in an additional increase in maternal mortality in India [16]. Thus, not only does it pose a significant public health risk, but also has social and economic impacts.

India is at a higher risk of the quick spread of Zika infection as numerous parallels exist between India and the countries most affected by the disease with respect to climate, distribution of mosquito species, and the prevalence of other arboviruses such as chikungunya and dengue; with a population that does not possess herd immunity to ZIKV [17]. The lack of proper sanitation practices in economically backward areas and large populations will make controlling the virus spread difficult. India might reach the middle ground with transmission levels high enough to maintain the virus, but not high enough to develop immunity. Around 465.7 million people in India would be at risk if a major ZIKV outbreak occurs [10, 18].

With the pandemic exposing the faltering health system, we cannot afford another outbreak in the country. Hence, creating awareness among the public about ZVD, vector control and preventing the disease are the best and the most cost-efficient options available. Long-term effects that affect the newborns will lead to a population that needs more medical and economic support adding on to the existing economic burden of the nation.

The aspect of mitigating Zika infections that is in practice in India is limited to mitigating the vector, and as such, there are a number of other avenues which can be explored to decrease the Zika risk significantly. These include actions at the topmost level, such as legislation to actions involving the lowermost rungs of the healthcare system, such as accredited social health activists (ASHA) workers, who can play a key role in disseminating valuable information to the most vulnerable population groups.

A proper protocol can be developed by the government for diagnosis, treatment and most importantly preventive actions; allocating appropriate funds for each aspect. Awareness about the disease among pregnant mothers should be prioritized and asked to take necessary precautions against being infected with ZIKV to prevent congenital deformities and other complications during pregnancy [19]. This can be achieved much quickly with the help of the ASHA workers, who also visit pregnant women to provide them with basic antenatal care, and thus help in reaching the innermost areas of the country.

Necessary precautions like minimizing bare skin exposure, mosquito proofing houses by installing screens on doors and windows, preventing the stagnation of water around the house, using protective nets around the bed and use of EPA-approved insect repellents in the areas affected by ZIKV are to be advised [20].

If a pregnant lady is diagnosed with ZVD, the mother should be advised to get an ultrasound scan to rule out abnormalities in the fetus. Two vaccines are in development in Phase II under Bharat Biotech for ZVD [21]; however, all the resources were diverted as a result of the pandemic to deal with COVID- 19.

Furthermore, identification of cases is through RT-PCR test of the blood samples of the infected [13]. In India, only those who test negative for dengue as well as chikungunya are being tested for ZIKV as the disease is not endemic. Hence, we do not know who acts as carriers of the disease owing to the lack of testing and asymptomatic carriers.

To prevent the virus from entering the endemic population of viruses in the country, we must be vigilant in detecting the cases as early as possible, especially at this crucial time where more breeding sites for the Aedes spp. are made available due to the monsoon season [22]. However, due to the pandemic, fewer resources are being diverted for creating awareness among the general public about ZVD. The health system has been concentrating on treating COVID-19 with a relatively decreased emphasis on other diseases including ZVD. Also, healthcare workers would not easily diagnose a patient presenting with ZVD, especially in the area’s endemic to other arboviral infections with similar symptoms, which typically peak during the monsoon season. Necessary precautions like minimizing bare skin exposure, mosquito proofing houses by installing screens on doors and windows, preventing the stagnation of water around the house, using protective nets around the bed and use of EPA approved insect repellents in the areas affected by ZIKV are to be advised. Other measures like public health education about the condition, legislative measures at the state, district and village levels to be implemented to prevent the breed of vectors. The only certain measure to prevent the disease from posing a major threat is to prevent the disease from spreading utilizing every means possible. This is in the hands of the people at the very grassroot level.

Thus, the situation is currently like a tightrope walk: the balance depends on adequate public health measures along with cooperation from the public. Each individual will have to do his/her part and maintain vigil to overcome the situation.

## Data Availability

Not applicable.

## Abbreviations

ZVD: Zika virus disease
ZIKV: Zika virus
WHO: World Health Organization
COVID-19: Coronavirus disease 2019
ASHA: Accredited social health activists
